# Identification of host DEAD-box RNA helicases that regulate cellular tropism of oncolytic Myxoma virus in human cancer cells

**DOI:** 10.1038/s41598-017-15941-1

**Published:** 2017-11-16

**Authors:** Masmudur M. Rahman, Eugenie Bagdassarian, Mohamed A. M. Ali, Grant McFadden

**Affiliations:** 1 The Biodesign Institute; Center for Immunotherapy, Vaccines, and Virotherapy; Arizona State University, Tempe, AZ 85287-5401 USA; 2 UMR 1161 Virology ANSES, INRA, ENVA, National veterinary school, 94700 Maisons-Alfort, France; 3 Department of Biochemistry, Faculty of Science, Ain Shams University, Abbassia, 11566 Cairo, Egypt

## Abstract

Myxoma virus (MYXV), a Leporipoxvirus, is being developed as an oncolytic virotherapeutic for the treatment of a variety of human cancers. MYXV tropism for human cancer cells is largely mediated by intracellular signaling networks that regulate viral replication or innate antiviral response pathways. Thus, MYXV is fully or partially permissive for the majority of human cancer cells that harbor defects in antiviral signaling, but a minority are nonpermissive because the virus infection aborts before its completion. To identify host factors relevant for MYXV tropism in human cancer cells, we performed a small interfering RNA (siRNA) library screen targeting the 58 human DEAD-box RNA helicases in two permissive human cancer cells (HeLa and A549), one semi-permissive (786-0), and one nonpermissive cell line (PANC-1). Five host RNA helicases (DDX3X, DDX5, DHX9, DHX37, DDX52) were inhibitory for optimal replication and thus classified as anti-viral, while three other cellular RNA helicases (DHX29, DHX35, RIG-I) were identified as pro-viral or pro-cellular because knockdown consistently reduced MYXV replication and/or required metabolic functions of permissive cancer cells. These findings suggest that replication of MYXV, and likely all poxviruses, is dramatically regulated positively and negatively by multiple host DEAD-box RNA helicases.

## Introduction

MYXV is the prototypic member of the Leporipoxvirus genus of Poxviridae family of viruses, which causes myxomatosis disease in European rabbits, but is utterly non-pathogenic for all other non-leporid species. Although MYXV exhibits a very narrow host range in nature, it has been shown to productively infect various classes of human cancer cells in culture^1^. This selective tropism occurs both *in vitro* and *in vivo* within tumor tissues of either mouse or human origin, and has led to MYXV being developed as a potential oncolytic virotherapeutic for various classes of human cancer. For example, in several preclinical *in vivo* cancer models MYXV is potently oncolytic for various distinct classes of cancers, such as pancreatic cancer, glioblastoma, ovarian cancer, melanoma, lung cancer and hematologic malignancies^2–4^.

The productive infection of human cancer cells by MYXV relies on the ability of the virus to bind, enter and successfully complete the viral replication cycle to create infectious progeny virus. Although a small number of cancer cell lines have been identified that cannot bind MYXV^5^, the vast majority of transformed cells tested to date permit binding of the virus, entry, virion uncoating, and launch of at least the early stages of the viral replication cycle. Unlike rabbit cells, where MYXV is able to overcome essentially every aspect of both intrinsic and induced cellular antiviral barriers, the productive replication in human cancer cells largely rely on whether the virus is able to successfully overcome the diverse innate cellular barriers^6^. MYXV ability to selectively kill human or mouse cancer cells and not their normal primary somatic cell counterparts largely depends on multiple contributing factors, about which much remains to be elucidated. Several of the known factors that have been identified so far include: 1) most cancer cells lack the full complement of synergistic antiviral responses to the combination of normal type I Interferon (IFN) plus tumor necrosis factor (TNF), and many harbor defects in either pathway alone^7^; 2) some cancer cells possess excessive levels of endogenously activated protein kinase B (PKB), also known as AKT, which pro-actively facilitates optimal MYXV replication^8^; 3) cellular tumor suppressor genes like p53, ataxia-telangiectasia mutated (ATM) and retinoblastoma (Rb) can also alter/regulate the tropism of MYXV in human cancer cells^9^; 4) the ability of MYXV to inhibit cellular antiviral signaling pathways, such as those mediated by Protein Kinase R (PKR), are essential for MYXV replication in diverse human cancer/transformed cells^10,11^. Thus, it does seem clear that selective cancer cell tropism of MYXV is tied to the ability of the infecting virus to effectively manipulate the signaling environment of the infected cell, unless the target pathway is already compromised by the transformed state, and the outcome is thus largely independent of the origin of the tumor tissues from where the cancer cells were derived. For the same reason, cancer cells from many other non-rabbit species, such as rodent, canine or feline, are also fully permissive to MYXV infection, even though none of these are permissive hosts for infection by MYXV^12–14^.

The cellular superfamily of RNA helicases, also known as DExD/H-box helicases, are involved in every aspect of RNA metabolism^15,16^. However, in recent years, their involvement has been identified in an increasing number of other cellular functions such as: innate immune responses against pathogens, oncogenesis, and inflammatory diseases^17,18^. Emerging evidences suggest that mutations in multiple RNA helicase genes are frequently associated with oncogenesis, for example, mutations in DDX41 were identified from 3% of MDS/AML patients^19^. RNA helicase A/DHX9 also plays a role in cancer and inflammatory diseases and thus making it a potential therapeutic target^20^. DDX3, DDX5/p68, DDX17/p72 have all been implicated in human malignancies, although very few primary mutations have been identified in these RNA helicases^21,22^. Apart from direct mutations, increased levels of RNA helicases, for example EIF4A1 has been detected in multiple cancers^23^.

Some members of DEAD-box RNA helicases are also required for permissive replication of human viral pathogens like human immunodeficiency virus type 1 (HIV-1), hepatitis C virus (HCV), Dengue, and West Nile Virus, and are often target host factors for antiviral drugs^24^. For example, RNA helicases DDX1, DDX3, DDX5, DDX17, DDX21, and DDX56 are all involved in the regulation of HIV-1 replication^25^. On the other hand, some RNA helicases, such as RIG-I-like receptors (RLRs), are negative regulators of virus replication by acting as cytoplasmic viral nucleotide sensors that trigger innate signal pathways that induce the innate interferon responses against diverse viruses^26^. Depending on the context, some members of the RNA helicase family exhibit either pro-viral or anti-viral roles, for example DDX3 and DHX9 are the most studied helicases for both functions^20,27^. The influence of RNA helicases in poxvirus replication has not yet been studied in great detail. Earlier studies reported that RNA helicase DDX3 is a host target of vaccinia virus protein K7 host range factor^28,29^. Ectopic expression of DDX3 enhanced antiviral immune response by activation of interferon regulatory factor (IRF). In the case of MYXV, we previously identified DHX9 / RNA helicase A (RHA) as a host protein that physically interacts with the viral host range factor M029, which also interacts with host cellular PKR, modulating MYXV tropism in a number of tested human cancer cells^10^.

Based on our previous studies, MYXV infection and replication in human cancer cells can be classified as fully permissive (high levels of infectious progeny, up to 100 FFU/cell), semi-permissive (lower levels infectious progeny, generally less than 10 FFU/cell) and nonpermissive (no infectious progeny)^8,30,31^. However, to date very few cellular factors that restrict or permit MYXV replication in transformed human cells have been identified. Cellular AKT, for example can restrict MYXV replication based on its phosphorylation status and the successful manipulation of AKT activation by the MYXV-encoded protein M-T5, a host range factor^8^. By pharmacological manipulation of AKT phosphorylation, it was possible to significantly increase MYXV replication in some semipermissive/nonpermissive human cancer cells^31^. In an effort to identify additional host factors that either restrict or enhance MYXV replication in human cancer cells, a genome-wide small interfering RNA (siRNA) library screen has been performed^32^. This screen has reported identification of large set of genes that can either enhance or reduce MYXV replication in human MDA-MB231 breast cancer cells^32^. However, for large set of target genes, it is difficult to identify how viruses manipulate them and whether viral proteins are directly involved in their manipulation. Indeed, individual genome wide siRNA library screens for the same virus by different groups have identified a surprisingly low overlap between the studies^33^. Nevertheless, systemic functional genomic screens using siRNA libraries have identified new candidate host proteins with potential roles in the replication of multiple viruses, including numerous human pathogens and DNA viruses^34^. In this study, we have screened a small siRNA library that targets the known cellular RNA helicases (58 genes) in human cancer and transformed cell lines of either the permissive or nonpermissive phenotype. We have identified five anti-viral and three pro-viral RNA helicases whose knockdown depletion significantly enhanced or reduced MYXV replication, respectively in the different classes of human cancer cells.

## Results and Discussion

The first round of screening of the full complement of human RNA helicases that altered MYXV gene expression was performed in 96 well plates in HeLa cells in triplicate. Our previous experience was that HeLa cells can be reliably and efficiently transfected with siRNAs^10^. We initially infected the cells with GFP-expressing MYXV and observed the fluorescence intensity using fluorescence microscopy. In addition, we also used a quantitative approach to quantify the levels of viral gene expression after RNA helicase siRNAs transfection using a reporter Fluc-expressing MYXV, vMyx-Fluc. Combining these two approaches we were able to identify candidate RNA helicases that enhanced or reduced MYXV gene expression in human cancer cells (Supplementary Table S1 and Supplementary Fig. S2). This primary screening allowed us to reduce the number of candidate siRNAs for more detailed analyses of candidate target RNA helicases down to 24 from 58.

**Figure 1:**
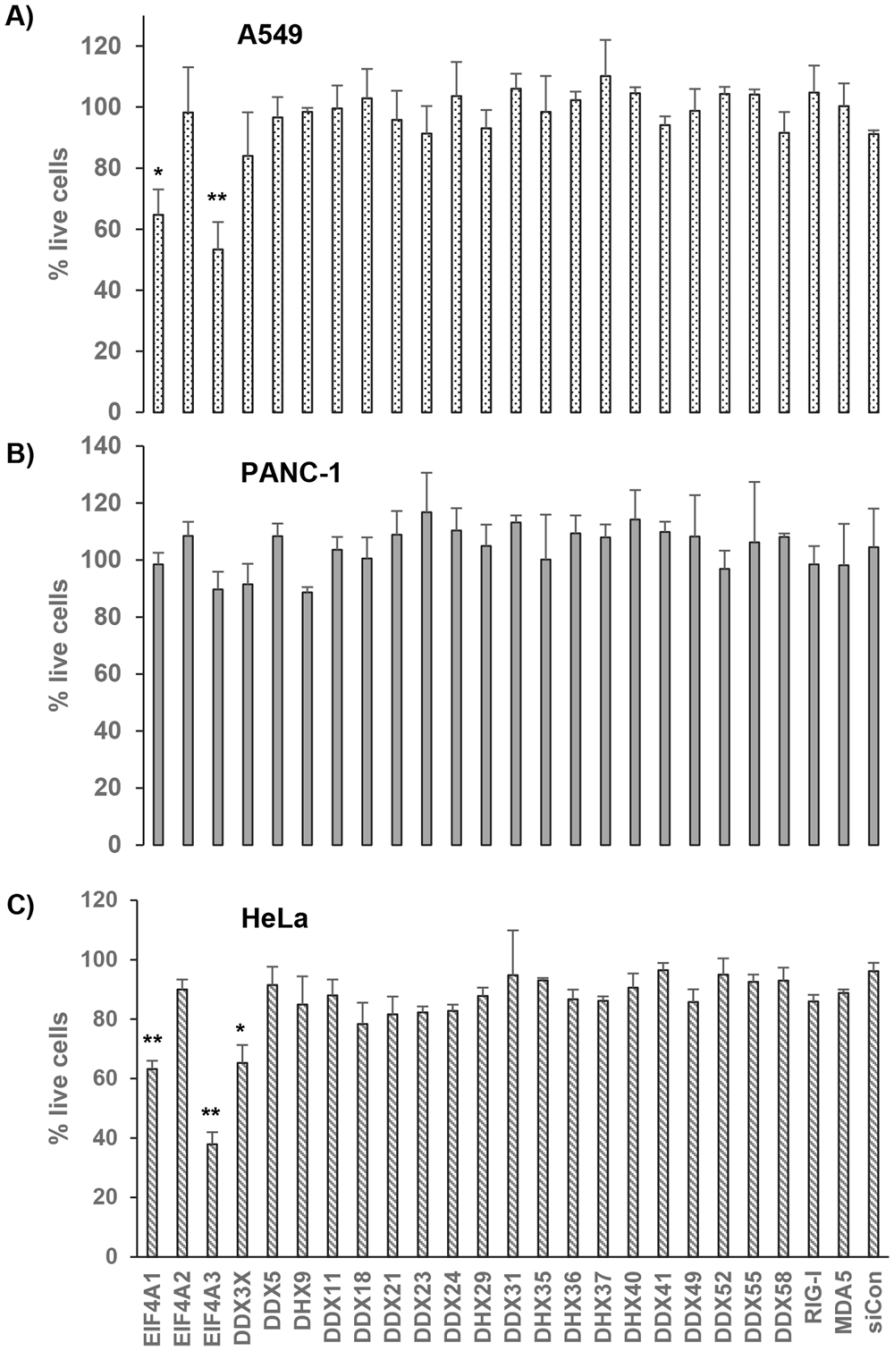
Viability assay using MTT. A549 (**A**), PANC-1 (**B**) and HeLa (**C**) cells were plated in 96 well plates and transfected with indicated siRNAs using RNAiMAX. At 72 h post-transfection, the cellular viability was measured using MTT assay reagents. The experiment was performed in triplicate and values represent mean ± SD. Statistically significant differences from control non-targeting siRNA (siCon)-treated cell are indicated.

In order to test whether the siRNAs for selected RNA helicases after primary screening had effect on cell viability which also could nonspecifically alter virus replication, we performed a cell viability assay of siRNA-transfected cells by MTT assay. This assay was performed in the absence of virus infection, to monitor the potential toxic effects of siRNA alone, where puromycin (10 µg/ml) was used as positive control to fully inhibit host cell translation (data not shown). We performed the assay in PANC-1 (nonpermissive for MYXV), A549, and HeLa cell lines (both permissive) as representative human cancer cell lines (Fig. 1). A549 and HeLa cell lines are known to support replication of MYXV similarly to that observed in rabbit cells, while PANC-1 cells do not support productive MYXV replication to any detectable level^10,35^. The MTT assay results indicate that none of the siRNAs showed dramatic reduction in cell viability, compared to that observed with puromycin. However, transfection of siRNAs for two of them, namely EIF4A1 and EIF4A3, significantly reduced the viability of both A549 and HeLa cells (Fig. 1A and C). In contrast, none of the tested RNA helicase siRNAs significantly reduced the viability of PANC-1 cells in this assay (Fig. 1B).

**Figure 2:**
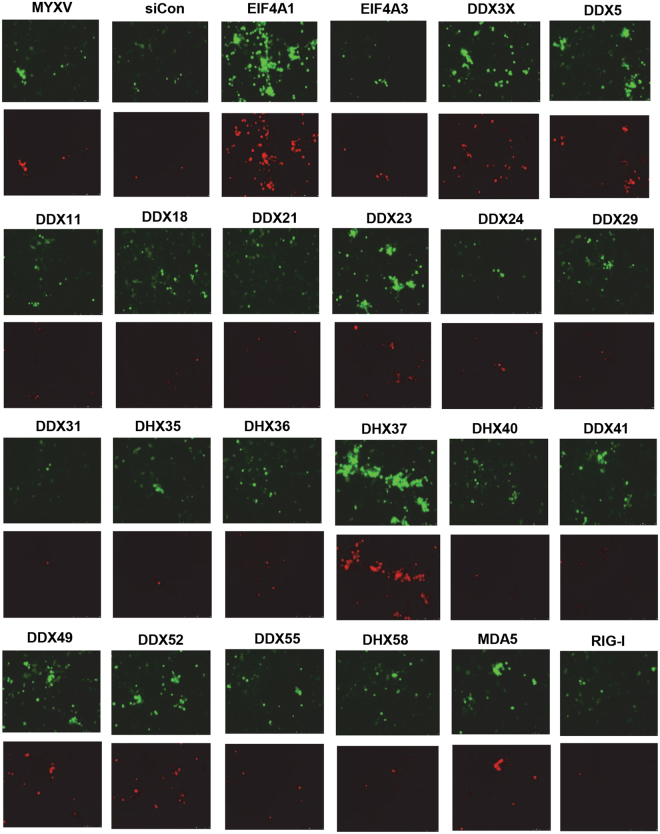
Effects of selected RNA helicase siRNAs on early and late gene expression of MYXV in non-permissive PANC-1 cells. PANC-1 cells were plated in 48 well plates, transfected with control non-targeting siRNA (siCon) or siRNAs targeted to the indicated genes. After 48 h, the cells were infected with vMyx-GFP-TdTomato at MOI of 1 FFU/cell and virus-containing media was replaced with fresh media after 1 h of virus adsorption. Images showing GFP (top panels) and TdTomato (bottom panels) of the siRNA-transfected and virus-infected PANC-1 cells were taken 72 h after infection using a Leica fluorescence microscope.

**Figure 3:**
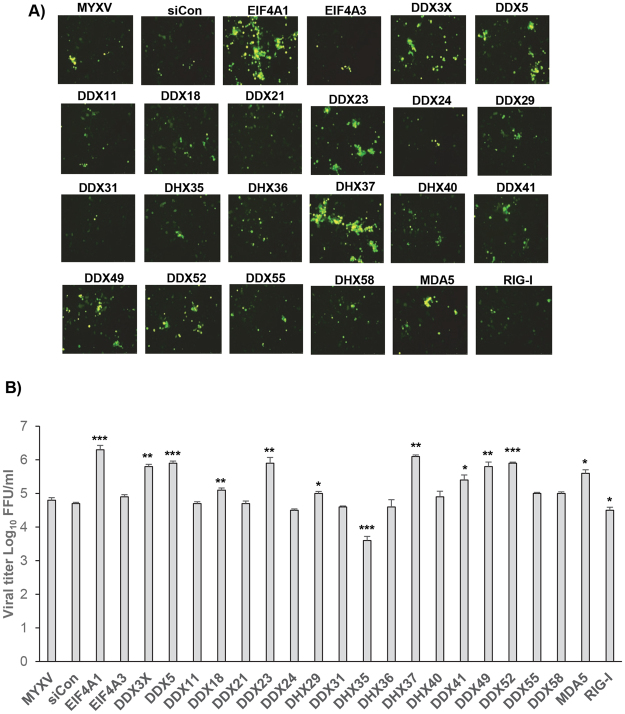
Effects of selected RNA helicase siRNAs on replication of MYXV in non-permissive PANC-1 cells. PANC-1 cells were seeded, transfected with siRNAs, and infected with vMyx-GFP-TdTomato virus as described in Fig. 2. The cells were harvested at 72 hpi to determine progeny virus formation by titration assay on permissive RK13 cells. (**A**) Overlay showing the cells expressing both GFP and Tdtomato (yellow color); (**B**) The virus titers were determined in triplicate following serial dilution onto RK13 cells. Statistically significant differences in comparison to infection with MYXV alone are indicated; *p < 0.05; **p < 0.01; ***p < 0.001.

The selected RNA helicase siRNAs after the first round of screening with HeLa cells were assessed again in multiple cell lines to confirm their effect on MYXV replication. This second round of screening was performed in 48 well plates using PANC-1, HeLa and A549 cells. In this assay, after siRNA transfection and infection with reporter vMyx-GFP-TdTomato virus, the cells were first monitored for the expression of GFP under early/late viral promoter and TdTomato under late viral promoter. To further confirm the sequential stages of MYXV replication, the cells were harvested at the indicated time points for virus titration. The nonpermissive PANC-1 cells were infected with an MOI of 1.0 after siRNA transfection and monitored for the expression of the reporter viral fluorescence proteins at different time points after infection and finally the progeny virus was titrated in permissive RK13 cells. In PANC-1 cells, infection with MYXV alone (no siRNA) revealed reduced number of TdTomato expressing cells than early GFP expressing cells after 72 h (Fig. 2), which was confirmed by overlay of GFP and TdTomato images, showing yellow colored cells (Fig. 3A). This suggests that in most PANC-1 cells there was a block in late MYXV gene expression, which aborted virus replication and prevented progeny virus formation^35^. Results from the siRNA screening suggest that some of the RNA helicase knockdowns enhanced viral late gene expression as monitored by the TdTomato expression and foci formation (Figs 2 and 3A). When we assessed progeny virus formation, we observed increases in virus titer with certain RNA helicase knockdowns, suggesting a partial rescue of virus replication (Fig. 3B). More than a 10 fold (1 log_10_) increase in virus titer compared to virus infection alone or non-targeting siRNA (siCon) transfection was observed with siRNAs for EIF4A1, DDX3X, DDX5, DDX23, DHX37, DDX49, DDX52 RNA helicases. Interestingly, siRNA for DHX35, further reduced virus titer even in these nonpermissive PANC-1 cells compared to the controls (Fig. 3B). We also tested the same 24 RNA helicase siRNAs in HeLa and A549 cells, which are permissive to MYXV replication. The results indicate that in permissive HeLa and A549 cells, siRNA transfection and infection with an MOI of 1.0, the RNA helicase knockdown did not change virus titers as dramatically as in the nonpermissive PANC-1 cells (Table 1). However, for HeLa cells, even under these conditions, a significant increase in virus titer was observed with siRNAs for DDX5, DHX9 and DDX55 (Table 1). Unlike PANC-1 cells, in HeLa and A549 cells siRNAs for EIF4A1 and EIF4A3 reduced virus titer significantly, which is consistent with the reduction of cell viability with these siRNAs. In addition, we also observed reduction in virus titer with siRNAs for DDX23, DHX29, DHX35 and RIG-I in either PANC-1, HeLa, or A549 cells (Table 1). Thus, with our sequential screening strategy, we were able to further select a subset of siRNAs that can either enhance (DDX3X, DDX5, DHX9, DHX37, DDX52) or reduce (DHX29, DHX35, RIG-I) MYXV replication in human cancer cells.

**Table 1:** Effects of siRNA-mediated RNA helicase knock down on MYXV replication in different human cell lines.

siRNAs	Fold changes		
	PANC-1	HeLa	A549
EIF4A1	36.5***	−1, n.s.	−2.5*
EIF4A2	n.t.	−0.5, n.s.	0.3, n.s.
EIF4A3	0.5, n.s.	*n.p.v*.	*n.p.v*.
DDX3X	10.6**	−1.4*	0.3, n.s
DDX5	13.5***	1.8*	n.t.
DHX9	n.t.	1.1**	0, n.s.
DDX11	−0.2, n.s.	0.1, n.s.	0.6, n.s.
DDX18	1.3**	0.2, n.s.	0.3, n.s.
DDX21	−0.3, n.s.	0.4, n.s.	0.2, n.s.
DDX23	13.3**	*n.p.v*.	−0.2, n.s.
DDX24	−0.8, n.s.	0.3, n.s.	0.4, n.s.
DHX29	0.8*	−1**	−0.5, n.s.
DDX31	−0.3 n.s.	0.3, n.s.	0.5, n.s.
DHX35	*n.p.v*.	−1.7*	−0.2, n.s.
DHX36	−0.3, n.s.	0.4, n.s.	−0.1, n.s.
DHX37	19.7**	0.3, n.s.	0.9, n.s.
DDX40	0.6, n.s.	0.2, n.s.	0.3, n.s.
DDX41	3.1*	0.8*	0.3, n.s.
DDX49	10**	−1.2*	0.1, n.s.
DDX52	13.1***	0.4, n.s.	0.3, n.s.
DDX55	0.6*	1.8**	n.t.
DDX58	0.6, n.s.	1*	0.5, n.s.
MDA5	5.4*	0, n.s.	0.3, n.s.
RIG-I	−1*	n.t.	−1.1, n.s.
siCon	−0.2, n.s.	−1*	0.2, n.s.

*p < 0.05; **p < 0.01; ***p < 0.001; n.s., not significant; n.p.v, no progeny virus; n.t., not tested; RNA helicase siRNAs that enhanced MYXV titers are in **bold** and RNA helicase siRNAs that reduced MYXV titers are in *italic*.

**Figure 4:**
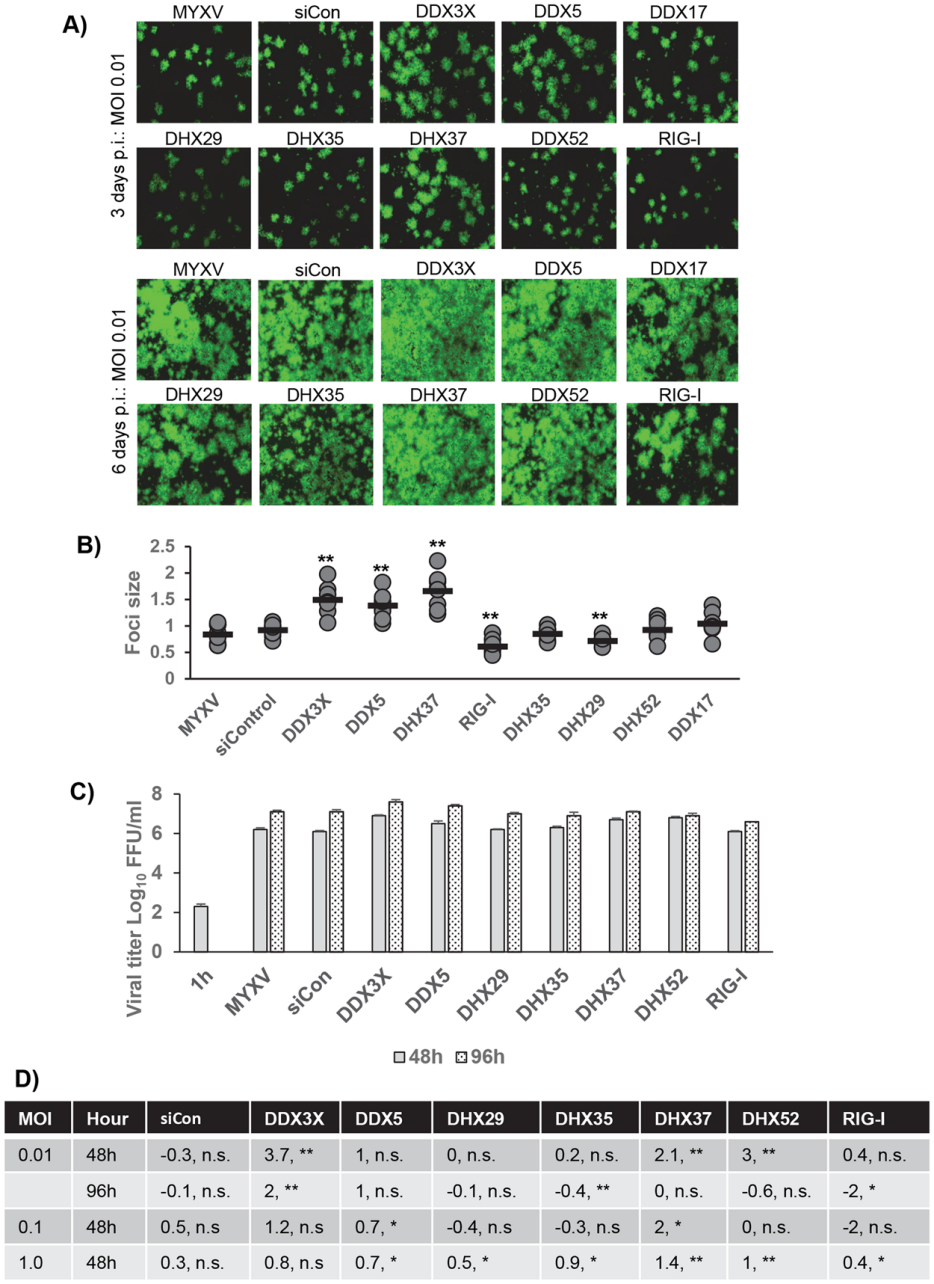
Effects of selected RNA helicase siRNAs on replication of MYXV in permissive human A549 cells. A549 cells were plated in 48 well plates, transfected with control siRNA or siRNAs targeted to the indicated RNA helicases. After 48 h the cells were infected with vMyx-GFP-TdTomato with the indicated MOI and virus-containing media was replaced with fresh media after 1 h of virus adsorption. The cells were harvested at the indicated time points to determine progeny virus formation by titration assay on permissive RK13 cells. Images of the siRNA-transfected and virus- infected A549 cells: (**A**) infected with MYXV at MOI of 0.01 FFU/cell and images were taken 3 days post infection (p.i.) (upper panels) or 6 days p.i. (lower panels); (**B**) foci size from images taken on 3 days post infection were measured using image J software and plotted. At least 8–10 foci were measured for each siRNA treatment. Statistically significant differences compared to infection with MYXV alone are indicated; **p < 0.01. (**C**) The virus titers after 48 and 96 hrs post infection with MOI of 0.01 are shown. (**D**) Table showing fold changes in virus titer after transfection of selected RNA helicase siRNAs and infection with different MOIs compare to the infection with MYXV alone. *p < 0.05; **p < 0.01; n.s., not significant.

The selected RNA helicases after this second round of screening, were further evaluated for their ability to influence the replication of MYXV in human cancer cells. In this case, we tested different MOIs (0.01, 0.1 and 1.0) to monitor whether at low MOI in permissive cells, we can observe significant changes in virus titer, as we observed in PANC-1 cells (Figs 2 and 3). A549 cells were plated in 48 well plates, transfected with DDX3X, DDX5, DDX17 (as a control for its reported interaction with DDX5^36^), DHX29, DHX35, DHX37, DDX52 and RIG-I RNA helicase siRNAs and infected with different MOI to monitor foci formation and virus replication. The fluorescence images of foci were taken 3 and 6 days after infection with an MOI of 0.01 (Fig. 4A), which clearly demonstrate that DDX3X, DDX5 and DHX37 enhanced the foci size significantly when compared to the control siRNA or infection with the virus alone. We have further measured the foci size on day 3 using image J software to compare the foci sizes among the different RNA helicase siRNAs treatment (Fig. 4B). We also observed a significant reduction in foci size with DHX29 and RIG-I helicases knockdowns. The cells were further monitored for 6 days, which clearly demonstrate that increased foci sizes was mediated by enhanced virus spread (Fig. 4A, bottom panels). We also measured the formation of infectious progeny virus after 48 and 96 h post infection with an MOI of 0.01 (Fig. 4C). The results indicated that the enhanced foci sizes corresponded also to increased virus titer (Fig. 4C). In A549 cells, virus titration at different MOIs was also performed (Fig. 4D). The results indicate that with MOI of 0.01, we observed maximum increase in virus titer with DDX3X, DHX37 and DHX52 compared to MOI of 0.1 and 1.0 (Fig. 4D).

**Figure 5:**
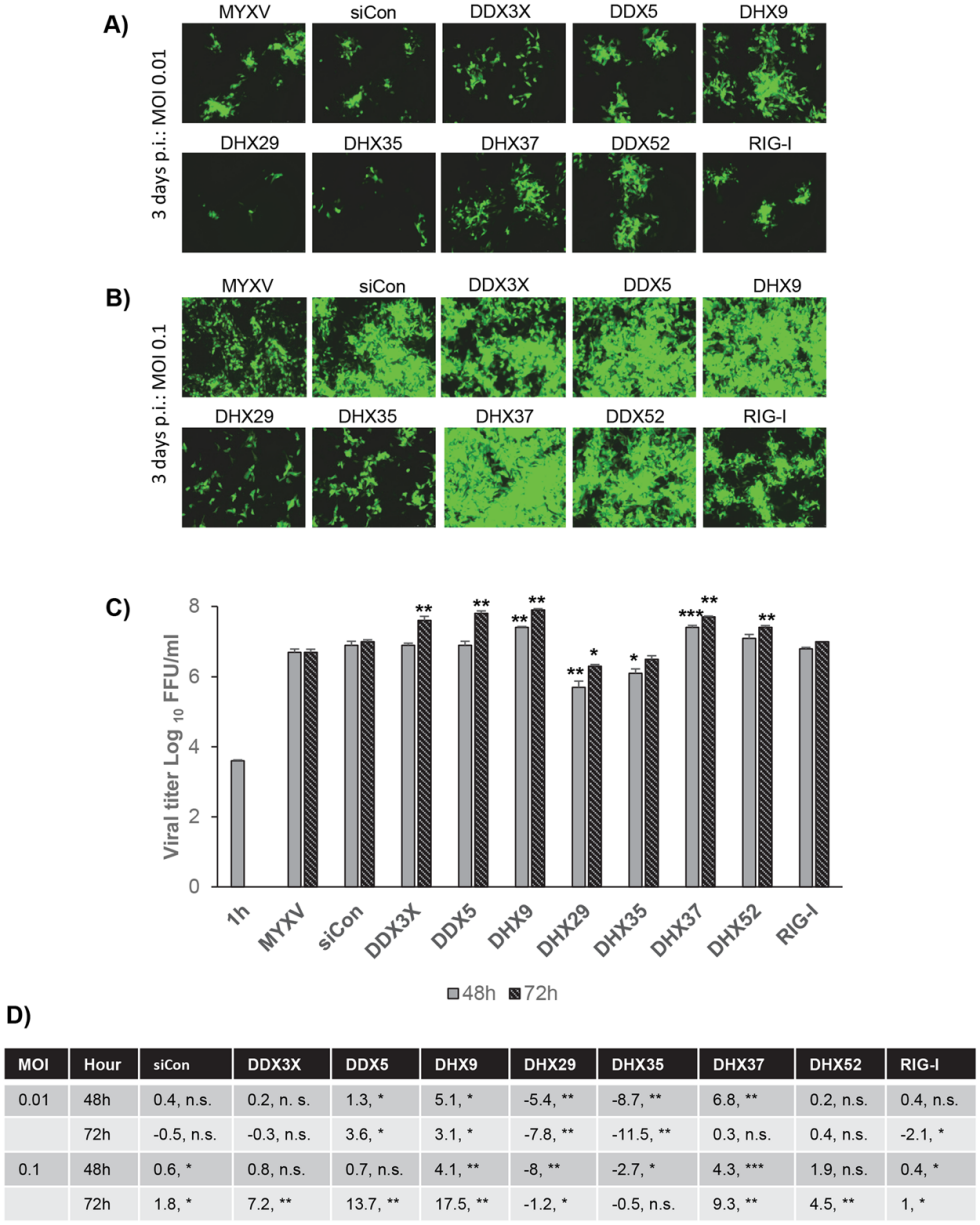
Effects of selected siRNAs on replication of MYXV in 786-0 cells. 786-0 cells were plated in 48 well plates, transfected with siCon or siRNAs targeted to the indicated RNA helicases. After 48 h, the cells were infected with MYXV at MOI of 0.01 or 0.1 FFU/cell for 1 h and replaced with fresh media. At the indicated time points the cells were harvested to determine infectious virus production by titration assay on permissive RK13 cells. Images of the siRNA-transfected and virus-infected 786-0 cells: (**A**) infected with MYXV at MOI of 0.01 FFU/cell and images taken 3 days post infection, or (**B**) infected with MYXV at MOI of 0.1 FFU/cell and images were taken 3 days post infection. (**C**) The virus titers after 48 and 72 hrs post infection with MOI of 0.1 are shown. (**D**) Table showing fold changes in virus titer after transfection of selected RNA helicase siRNAs and infection with different MOIs compared with the infection with MYXV alone. *p < 0.05; **p < 0.01; ***p < 0.001; n.s., not significant.

To further confirm the selected RNA helicases knockdown effects on MYXV replication, a human renal cancer cell line 786-0, which can be semi-permissive or nonpermissive depending upon the activation status of AKT and the presence of the M-T5 viral host range protein, was tested. 786-0 cells were classified as type II cells based on the level of endogenous phosphorylated-AKT level and restriction of M-T5 KO MYXV replication^8^. However, WT-MYXV has no defect in foci and progeny virus formation in these cells. The 786-0 cells were seeded in 48 well plates and transfected with the DDX3X, DDX5, DHX9, DHX29, DHX35, DHX37, DDX52 and RIG-I RNA helicase siRNAs. After 48 h of transfection, the cells were infected with the GFP-expressing MYXV. Since 786-0 is semi-permissive to MYXV replication two different MOIs 0.01 and 0.1 were used. The infection progression was observed using fluorescence microscope and recorded after 3 days post infection (Fig. 5A and B). Again, in 786-0 cell line at an MOI of 0.01 (Fig. 5A), knockdown of RNA helicases like DDX3X, DDX5, DHX9, DHX37 and DDX52 enhanced the foci size while DHX29, DHX35 and RIG-I reduced the foci size compared to the control siRNA or infection with the virus alone. This effect on virus replication and spread was also observed when the cells were infected with MOI of 0.1 (Fig. 5B). We also measured the progeny virus formation under these different infection conditions (Fig. 5C and D). The results indicate that, in 786-0 cells infection with MOI of 0.1 increased MYXV titer more than 1 log with knockdown of RNA helicases DDX5, DHX9 and DHX37 compared to infection with MYXV alone (Fig. 4D). Like A549 cells, RNA helicases DHX29 and DHX35 knockdown also reduced progeny MYXV titer in 786-0 cells (Fig. 5D).

**Figure 6:**
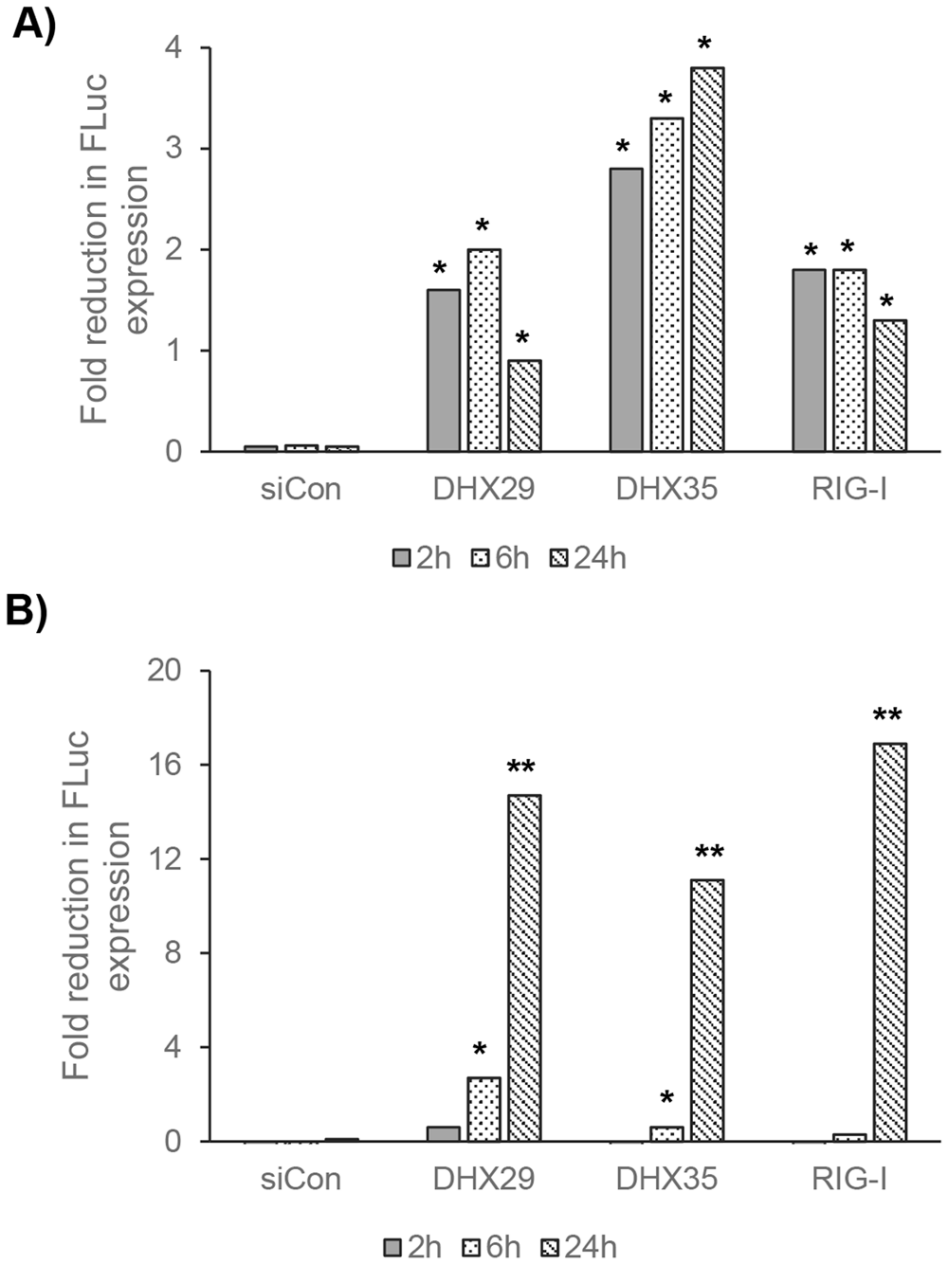
Knockdown of RNA helicases DHX29, DHX35 and RIG-I reduces the expression of MYXV early and late proteins. (**A**) 786-0 and (**B**) A549 cells were transfected with siCon or siRNAs targeted to the indicated RNA helicases. After 48 h, the cells were infected with vMyx-FLuc at MOI of 1 FFU/cell for 1 h and replaced with fresh media. Cells were then collected at the indicated time points and processed for luciferase assay. The assays were done in triplicate. Fold reduction in Fluc expression was calculated from the samples infected with MYXV alone. *p < 0.05; **p < 0.01.

We also tested whether the reduction or increase in virus titers was due to the changes in viral gene expression after RNA helicase knockdowns. We measured the expression of FLuc protein at early and late times of infection (2 h, 6 h and 24 h) in both 786-0 and A549 cells. In 786-0 cells, knockdown of RNA helicases DHX29, DHX35 and RIG-I significantly decreased FLuc expression when compared to the virus infection alone only after 2 h post infection, while the control siRNA did not show this effect (Fig. 6A). This suggests that these RNA helicases might have a role during early gene expression of MYXV in human cells. These RNA helicases also reduced FLuc expression in A549 cells, however, unlike 786-0 cells, the reduction was significant starting from 6 h post infection (Fig. 6B). On the other hand, for the DDX3X, DDX5, DHX9, DHX37 and DHX52 knockdowns, which we have shown to result in increased virus titers in previous assays, we did not observe an early increase in FLuc expression. However, due to high levels of virus replication, we observed an increase in Fluc expression at late time of virus infection (as shown in Fig. S2), this suggests these helicases may not have direct role in the suppression of virus gene expression.

**Figure 7:**
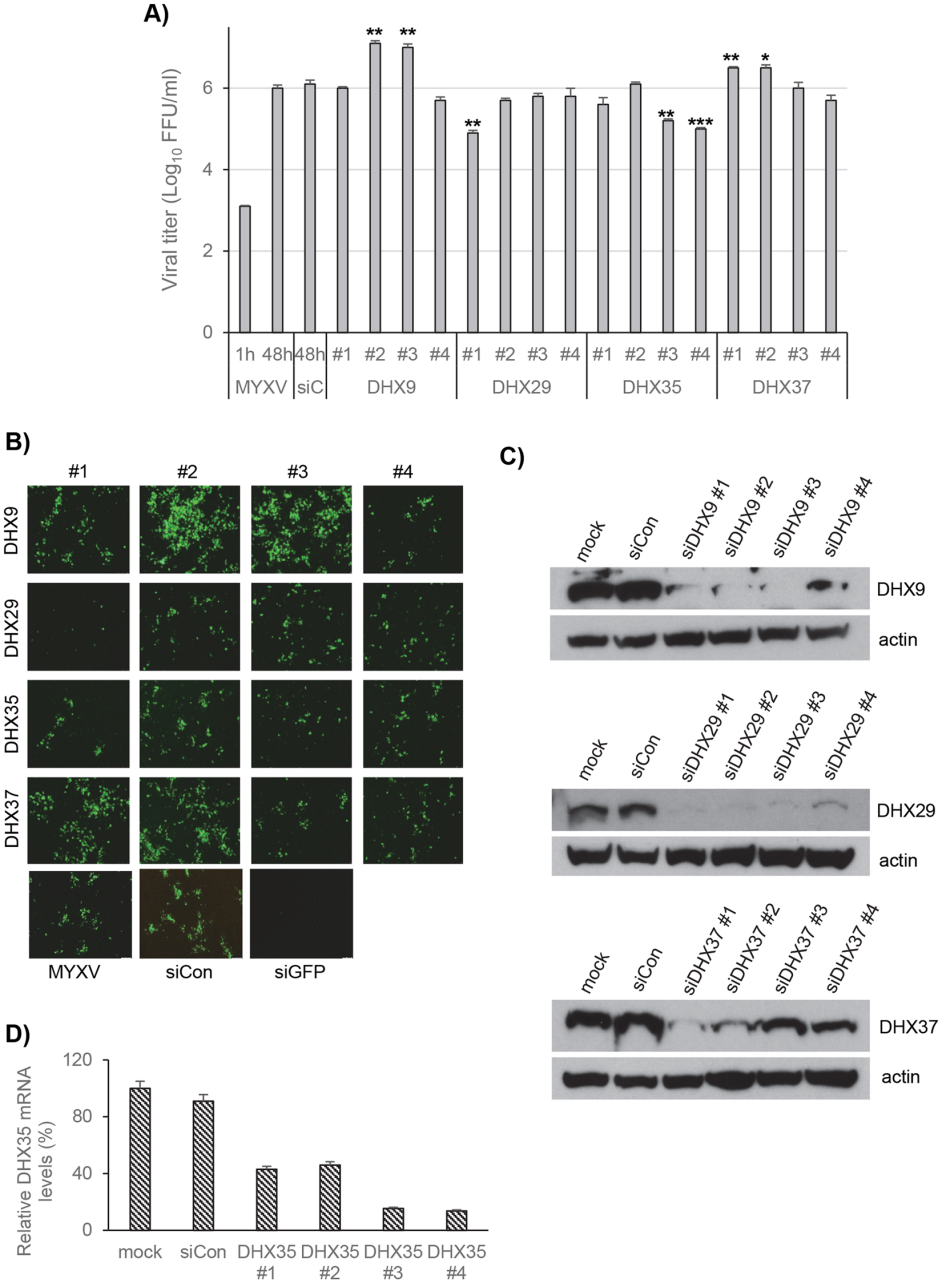
Effects of individual siRNAs on replication of MYXV in 786-0 cells. 786-0 cells were plated in 48 well plates, transfected with siCon or individual siRNAs targeted to the indicated RNA helicases. After 48 h, the cells were infected with MYXV at MOI of 0.01 FFU/cell for 1 h and replaced with fresh media. The cells were harvested at 48 hpi to determine progeny virus formation by titration assay on permissive RK13 cells. (**A**) The virus titers were determined in triplicate following serial dilution onto RK13 cells. (**B**) Images showing expression of GFP after 2 days post infection. (**C**) Western blot analysis of DHX9, DHX29 and DHX37 RNA helicases from 786-0 cells to detect endogenous protein levels after transfection of individual siRNAs; actin as loading control. (**D**) Relative quantification of DHX35 RNA helicase gene expression after siRNA transfections. *p < 0.05; **p < 0.01; ***p < 0.001.

To determine whether the results observed with the pooled siRNAs are specifically caused by knocked down of target genes and not by off-target effects, individual siRNAs against the RNA helicases of interest (in this case DHX9, DHX29, DHX35 and DHX37) were purchased from Dharmacon and tested again in 786-0 cells. Two siRNAs for DHX9 (DHX9 #2 and DHX9 #3) and DHX37 (DHX37 #1 and DHX37 #2) significantly enhanced progeny virus formation (Fig. 7A). Other two siRNAs for DHX9 and DHX37 had little or no effect on MYXV replication (Fig. 7A). The results are also confirmed by monitoring infection progression using fluorescence microscope (Fig. 7B). On the other hand, one siRNA for DHX29 (DHX29 #1) and two siRNAs for DHX35 (DHX35 #3 and DHX35 #4) significantly reduced progeny virus formation in 786-0 cells (Fig. 7A). Other siRNAs for DHX29 and DHX35 had little or no effect on MYXV replication. The results are also confirmed by monitoring infection progression using fluorescence microscope (Fig. 7B). This difference in effect of individual siRNAs against the same target may represent altered levels of knockdown. To assess individual siRNA-mediated knockdown, Western blot analysis was performed on lysates of siRNA transfected cells. Individual siRNAs for DHX9, DHX29 and DHX37 reduced respective RNA helicase expression at different level (Fig. 7C). There was little or no effect on control β-actin levels. However, in some cases (for example, DHX29) the knock down at the protein level did not correlate with the different effects of siRNAs observed on viral replication and progeny virus formation. We hypothesize that the critical level of helicases to become functional may vary and immunoblotting assay may not be sufficiently sensitive to observe those critical differences. The inability of DHX35 antibody to detect the correct size protein, a Western blot for DHX35 is not shown. However, using RT-PCR we were able to measure the level of DHX35 mRNAs after transfection of different siRNAs for DHX35 (Fig. 7D). To rule out the possibility of individual siRNA–induced cell death might have effects on viral replication, we performed cell viability assay (MTT). The results suggest that none of the siRNAs caused significant level of cell toxicity that can reduce viral replication (supplementary Fig. S3).

Our DEAD-box RNA helicases siRNA library screening in multiple human cell lines consistently identified three RNA helicases (DHX29, DHX35 and RIG-I) for which the knockdown reduced MYXV gene expression and progeny virion production, suggesting that they have a potential proviral role that enhances MYXV replication in human cancer cells. So far, there are no studies reporting that these three RNA helicases are involved in replication for any virus (Fig. 7). Among these, RNA helicase RIG-I is by far the most studied and it is involved in nucleic acid sensing and activation that triggers anti-viral innate immune responses leading to type I IFNs production^26,37^. Nevertheless, in our screenings, RIG-I knockdown consistently reduced MYXV gene expression and replication in multiple human cancer cell lines. At this stage, it is difficult to predict how RIG-I knockdown might decrease MYXV gene expression, but one future goal is to understand the mechanisms of this apparent proviral effect of RIG-I in human cancer cells in greater detail. Interestingly, in primary human macrophages, MYXV is indeed sensed by RIG-I and functions as an antiviral sensor that induces interferon beta and aborts the virus replication cycle^38^. The clear difference in functioning of RIG-I as an antiviral factor in primary human myeloid cells but as a proviral factor in at least some transformed human cancer cells is fascinating and requires greater scrutiny.

DHX29 has a cytoplasmic location and has been reported as a *bona fide* translation initiation factor^39,40^. It is possible that DHX29 plays a direct/indirect role in MYXV replication since poxviruses also replicate in the cytoplasm. Apart from its role in translation, DHX29 has also been reported to sense cytosolic nucleic acids together with RIG-I in human airway epithelial cells and fibroblasts^41^. In addition to the DHX29, several other RNA helicases have recently been identified and were found to be involved in the RLR-mediated type I IFN production after viral infection, for example, DDX3, DHX36, DDX60^42^. On the other hand, unlike for RIG-I or DHX29, very little is known about the function of DHX35. Our results indicate that DHX35 might also have a role in MYXV replication and possibly for other DNA viruses too.

**Figure 8:**
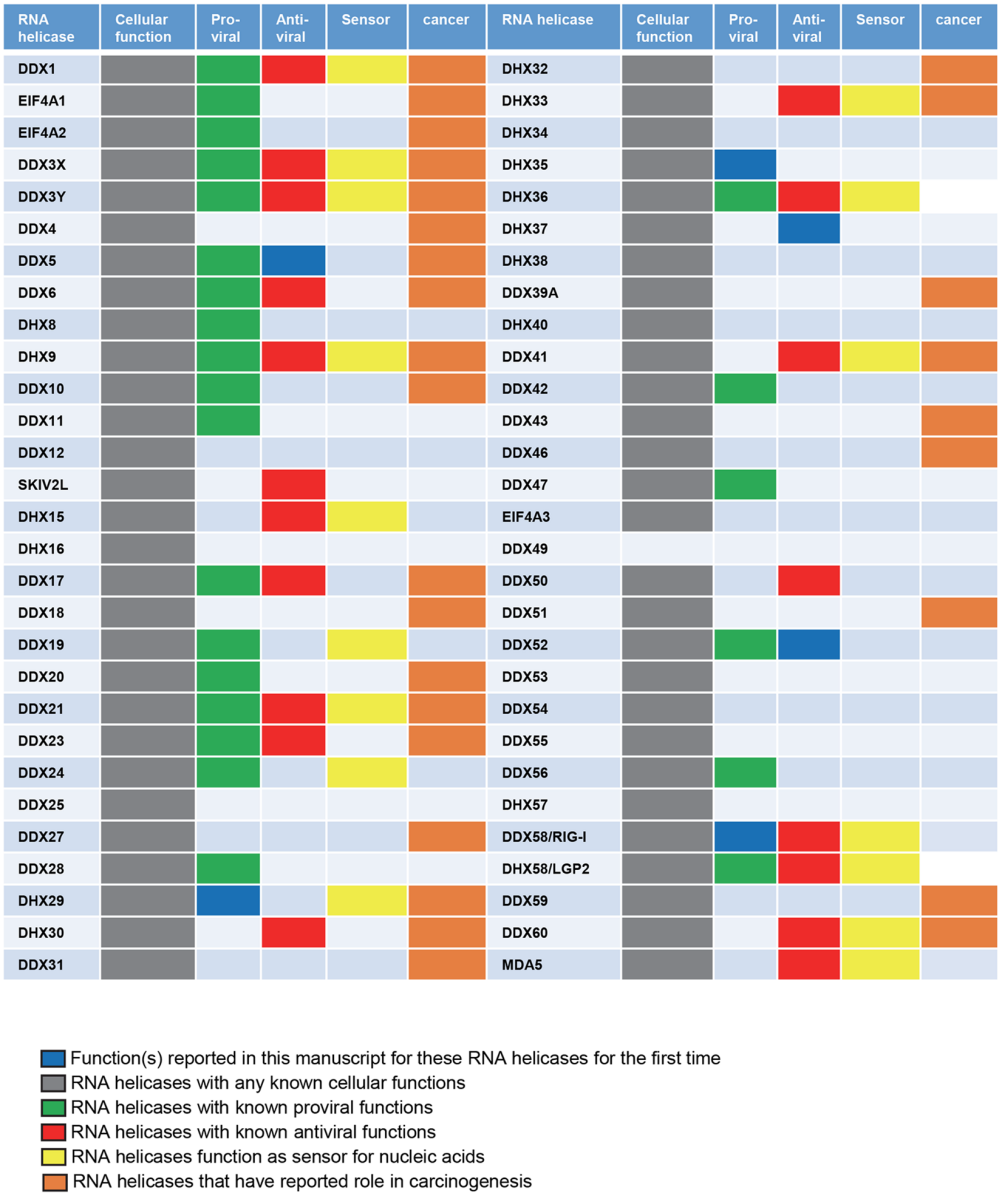
A list of the studied RNA helicases. RNA helicases with any known cellular functions are in gray, proviral functions are in green, antiviral functions are in red, sensing nucleic acids are in yellow, and oncogenesis are in brown. Functions of RNA helicases reported in this manuscript for the first time are in blue.

Our siRNA library screening also identified five RNA helicases, DDX3X, DDX5, DHX9, DHX37 and DDX52, of which the knockdown reproducibly increased MYXV replication in multiple human cell lines, suggesting that they all have potential roles either in antiviral responses or regulation of innate immune responses. These RNA helicases might not be directly involved in the inhibition of MYXV gene expression or replication, as observed by measuring poxvirus promoter driven expression of FLuc protein at early and late time points. We propose they function as direct or indirect regulators in the activation of antiviral signaling pathways. Among these RNA helicases, our study reports for the first time that DDX5, DHX37 and DDX52 might have anti-viral activities (Fig. 8). RNA helicases DDX5, DDX3X and DDX17 are reported to be involved in RNA virus replication such as HIV-1^43^, but our results indicate that they did not reduce MYXV replication. Our observation that the knockdown of RNA helicases DDX5, DDX3X and DHX52 enhanced MYXV foci size and virus spread supports their possible roles in the activation of antiviral innate responses. Among these RNA helicases, very little is known about the cellular function of RNA helicase DHX37. Both DDX3X and DHX9 are known to be involved in the induction of anti-viral responses^20,27^. Another RNA helicase, DDX21 has shown to induce innate immune responses against Dengue virus by translocation from nucleus to cytoplasm^44^. However, we have not observed any significant increase in MYXV replication in multiple human cancer cell lines after DDX21 knockdown. Based on these findings our future goal would be to identify which viral proteins target these RNA helicases and also to study whether they are directly involved in the process of activating antiviral immune responses. We are currently investigating the mechanisms of how these selected RNA helicases either enhance or reduce MYXV replication and also whether they have antiviral or proviral roles against other viruses. But for the purposes of developing MYXV as an oncolytic virotherapeutic against human cancer, these studies provide the first step in designing the next-generation virus constructs that extend the repertoire of potential cancer targets to even “nonpermissive” tumor cells that normally can resist infection by MYXV.

## Materials and Methods

### Cells, reagents, and viruses

Rabbit cell line RK13 (ATCC# CCL-37); and human cell lines HeLa (ATCC# CCL-2), A549 (ATCC# CCL-185), PANC-1 (ATCC# CRL-1469), and 786-0 (ATCC# CRL-1932) were cultured in Dulbecco minimum essential medium (DMEM; Invitrogen) supplemented with 10% fetal bovine serum (Atlanta Biologicals), 2 mM glutamine (Invitrogen) and 100 µg of penicillin-streptomycin (pen/strep; Invitrogen)/ml. All cultures were maintained at 37 °C in a humidified 5% incubator. vMyx-GFP (WT-MYXV that expresses GFP under poxvirus Syn E/L promoter), vMyx-GFP-Tdtomato (WT-MYXV that expresses GFP under Syn E/L promoter and Tdtomato under poxvirus p11 late promoter) and vMyx-FLuc (WT-MYXV that expresses firefly luciferase under poxvirus Syn E/L promoter) virus constructs, as described before, were used^45–47^. The virus stocks used were prepared using sucrose gradient purification as described before^48^.

### siRNA screening

The primary screening was conducted in 96 well plates using a custom RNA helicases (58) pooled siRNA library including non-targeting control (siCon) and siRNAs that target GFP and Firefly luciferase, as positive controls (ON-TARGETplus SMART pool siRNA from Dharmacon, Thermo Scientific). The primary screening was done in 96 well plates. The wells were seeded with the cells for 40–50% confluency, left over-night for adherence and then transfected with siRNA (20 nM) using the Lipofectamine RNAiMAX (Invitrogen) transfection reagent. After 48 h of transfection the cells were infected with the viruses and they were either observed under fluorescence microscope to monitor and record the expression of fluorescence proteins, or cells were lysed for luciferase assays. Second round of screening for RNA helicases and subsequent assays for virus replication were done in 48 well plates using the same siRNA transfection protocol.

### Virus replication assay

In 48 well plates, siRNA transfected cells were infected with different MOI of virus as described before^10^, the unbound virus was removed after 1 h and replaced with complete media. The cells were harvested at different times after infection and stored in −80 °C until all the samples were collected. The cells were lysed by 3 freeze thaw cycles and sonication. Dilutions of the lysate were plated on RK13 cells. After 48 h of incubation the foci were counted using fluorescence microscope.

### Luciferase assay

Firefly luciferase activity was measured to monitor viral gene expression in different cell lines under different treatment conditions. The assay was performed using 96 well plates. After cells were seeded and transfected with siRNAs as described before, F-Luc expressing virus, vMyx-FLuc with appropriate MOI was added directly to the cells. The media was changed after 1 h and harvested the cells at different time points after infection for the luciferase assay. The assay was performed using the luciferase reporter assay kit (Promega), following manufacturer instructions. Briefly, media was removed at the indicated time points, cells were washed with PBS once and added the lysis buffer. Lysis was done at RT for 15–20 mins and substrate was added and reading was taken immediately after adding the substrate using a microplate reader, Appliskan (Thermo Scientific).

### Cell viability assays

MTT (Promega, Madison, WI) based cell viability assays were performed according to the manufacturer’s instructions. In 96 well plate cells seeding and transfection of siRNAs was done as described above. After 72 h of siRNA transfection, MTT assays were performed. For all MTT assays, the values for siRNA transfected samples are reported as percent of live cells compared to the mock treated samples, which are considered 100% viable. MTT values reported are the average of triplicate samples.

### Western blot analysis

Western blot analysis was performed as described before^10^. Briefly, the mock or siRNA transfected cells were collected at different time points after transfection, washed with PBS and processed with RIPA lysis buffer. Equal amount of total proteins were used for Western blot analysis. The membranes were first probed with primary antibody, washed, incubated with secondary antibody, washed again and detected using the chemiluminescence substrate and exposure to X-ray film. The detection antibodies were as follows: mouse monoclonal antibodies against DHX9 and DHX29 (Santa Cruz Biotechnology); rabbit polyclonal antibodies against DHX37 (abcam, USA) and DHX35 (Novus Biologicals, USA); mouse monoclonal antibodies against actin (Ambion, Thermo Fisher Scientific).

### Real-Time polymerase chain reaction (qPCR)

The mock or siRNA transfected cells were harvested 48 h after siRNA transfection. Total RNA isolation, cDNA preparation and real-time PCR (qPCR) were performed based on the protocol described before^11^. The PCR reactions were run on an ABI 7300 qPCR machine under the following conditions: 95 °C for 10 min, followed by 40 cycles of 95 °C for 15 s and 60 °C for 1 min. Primers used for qPCR analysis are: human GAPDH (F: GTGGACCTGACCTGCCGTCT, R: GGAGGAGTGGGTGTCGCTGT), human DHX35 (F: AGCGAGGGGATCTTCGATTGA, R: AAATGCTAAAACGTCTCCGTCT). Amplification of genes was normalized to GAPDH amplification from the same sample and the fold changes after siRNA treatment was calculated relative to the mock treated sample.

### Statistical analysis

Data were expressed as mean ± *SD* and were analyzed by paired *t*-test. Significant differences were accepted at *p* < 0.05.

## Electronic supplementary material

supplementary information

Raw values from siRNA screen
