# Supplementary material for: Identification of host DEAD-box RNA helicases that regulate cellular tropism of oncolytic Myxoma virus in human cancer cells
Source: Sci Rep. 2017 Nov 16;7:15710. doi: 10.1038/s41598-017-15941-1 (PMC5691082; doi:10.1038/s41598-017-15941-1)
Supplement: Supplementary file 1 — supplementary information [file 41598_2017_15941_MOESM1_ESM.pdf]

Identification of host DEAD-box RNA helicases that regulate cellular tropism of oncolytic Myxoma virus in human cancer cells.

Masmudur M. Rahman[1], Eugenie Bagdassarian[2], Mohamed A.M. Ali[3] and Grant McFadden[1]*

[1]The Biodesign Institute; Center for Immunotherapy, Vaccines, and Virotherapy; Arizona State University; Tempe, AZ 85287-5401

[2]UMR 1161 Virology ANSES, INRA, ENVA, National veterinary school, 94700 Maisons-Alfort, France

[3]Department of Biochemistry, Faculty of Science, Ain Shams University, Abbassia, 11566, Cairo, Egypt.

*Corresponding author Email: grantmcf@asu.edu

Supplementary Table S1: RNA helicase siRNA primary screening

RNA helicases	FLuc activity	RNA helicases	FLuc activity
DDX1	n.s.	DHX32	n.s.
EIF4A1	+	DHX33	n.s.
EIF4A2	+	DHX34	-
DDX3X	+	DHX35	-
DDX3Y	n.s.	DHX36	+
DDX4	n.s.	DHX37	++
DDX5	++	DHX38	-
DDX6	n.s.	DHX39A	n.s.
DHX8	n.s.	DHX40	+
DHX9	++	DDX41	++
DDX10	n.s.	DDX42	n.s.
DDX11	+	DDX43	-
DDX12	n.s.	DDX46	n.s.
SKIV2L	n.s.	DDX47	-
DHX15	n.s.	EIF4A3	--
DHX16	n.s.	DDX49	+
DDX17	n.s.	DDX50	n.s.
DDX18	+	DDX51	n.s.
DDX19	n.s.	DDX52	++
DDX20	n.s.	DDX53	n.s.
DDX21	+	DDX54	n.s.
DDX23	-	DDX55	+
DDX24	-	DDX56	n.s.
DDX25	n.s.	DHX57	n.s.
DDX27	n.s.	DDX58/RIG-I	-
DDX28	n.s.	DHX58/LGP2	+
DHX29	--	DDX59	n.s.
DHX30	n.s.	DDX60	n.s.
DDX31	+	MDA5	+

n.s., not significant (more than 25% increase or decrease in FLuc activity); +, more than 25% increase in FLuc activity; ++, more than 50% increase in FLuc activity; -, more than 25% decrease in FLuc activity; --, more than 50% decrease in FLuc activity.

Supplementary Figure S2. Knockdown of RNA helicases alter the expression of MYXV early and late proteins. HeLa cells were transfected with siCon, as negative control; siRNA for F-Luc, as positive control and pooled siRNAs targeted to the RNA helicases. After 48h, the cells were infected with vMyx-FLuc at MOI of 1 FFU/cell for 1h and replaced with fresh media. The assays were done in triplicate.

Supplementary Figure S3. Viability assay for the selected individual siRNAs. 786-0 cells were plated in 96 well plates and transfected with indicated siRNAs using RNAiMAX. At 72h post-transfection, the cellular viability was measured using MTT assay reagents. The experiment was performed in triplicate and values represent mean ± SD.

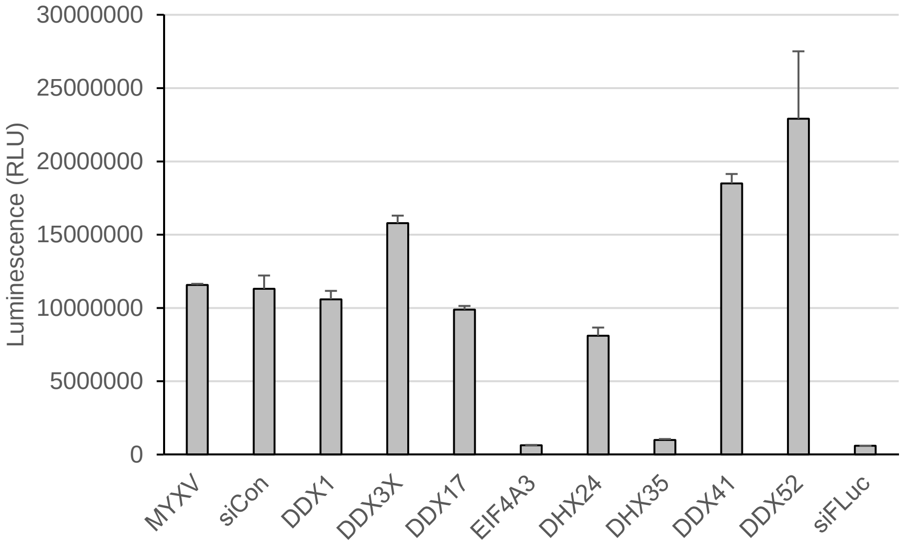

30000000
25000000
20000000
15000000
10000000
5000000
0
Luminescence (RLU)
MYXV
siCon
DDX1
DDX3X
DDX17
EIF4A3
DHX24
DHX35
DDX41
DDX52
siFLuc

Figure S2

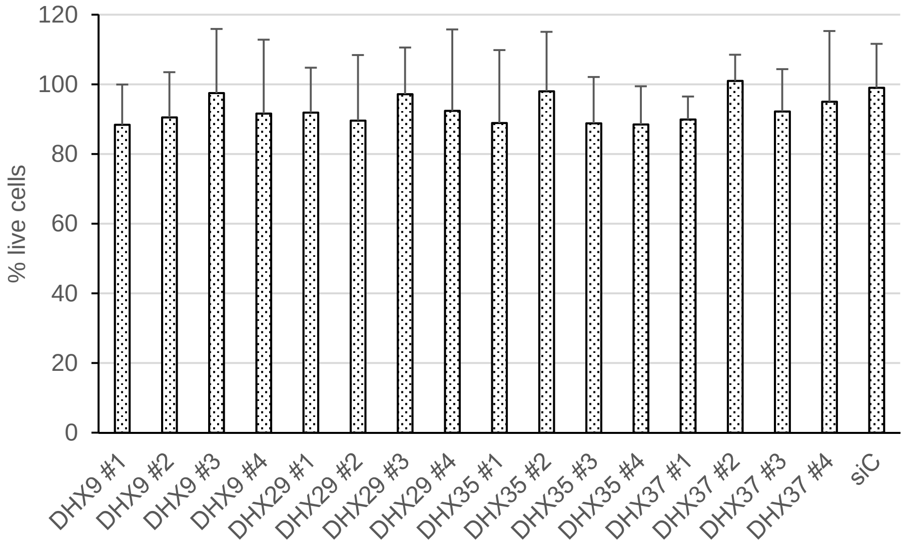

120
100
80
60
40
20
0
% live cells
DHX9 #1
DHX9 #2
DHX9 #3
DHX9 #4
DHX29 #1
DHX29 #2
DHX29 #3
DHX29 #4
DHX35 #1
DHX35 #2
DHX35 #3
DHX35 #4
DHX37 #1
DHX37 #2
DHX37 #3
DHX37 #4
siC

Figure S3

Values for supplementary table 1

siRNAs/control	test 1	test 2	test 3	Average	SD
MYXV	11598005	11473036	11625303	11565448	81186.69
SiCon	10640906	10959694	12340568	11313723	903446.2
DDX1	10368721	11238902	10145329	10584317	577786.1
EIF4A1	15347025	15600712	15198062	15381933	203582.1
EIF4A2	16709823	16292002	16358092	16453306	224594.8
DDX3X	15892376	16236901	15238290	15789189	507239.2
DDX3Y	12159348	11700473	12440873	12100231	373723.3
DDX4	11926031	12092045	12273401	12097159	173741.5
DDX5	20359627	20781568	21082391	20741195	363069.4
DDX6	9861038	9609823	10258332	9909731	326985.1
DHX8	12068391	12173081	11801832	12014435	191415.6
DHX9	27631804	26870021	28158035	27553287	647586.9
DDX10	11856303	10928623	11306824	11363917	466467.8
DDX11	15461839	15709722	15593002	15588188	124011.6
DDX12	12007935	11350972	11702371	11687093	328747.9
SKIV2L	10356283	10579024	11057941	10664416	358538.5
DHX15	12781035	12508467	13052584	12780695	272058.7
DHX16	11839171	11200845	12101372	11713796	463169.9
DDX17	9654821	9838276	10158376	9883824	254848.8
DDX18	17271340	17846721	17406349	17508137	300892.6
DDX19	13159062	12903782	13540128	13200991	320238.3
DDX20	12368032	11928014	11692104	11996050	343061.7
DDX21	15831263	16291003	15724083	15948783	301177.3
DDX23	8162971	83097403	7910384	33056919	43336514
DDX24	8741703	7724747	7852738	8106396	553901.3
DDX25	9806153	10293007	9690485	9929882	319749.6
DDX27	14090281	13574028	13400836	13688382	358665.8
DDX28	12061336	11682034	12203856	11982409	269715.9
DHX29	3124318	3804812	3381084	3436738	343643.8
DHX30	10932860	10491387	11203409	10875885	359414
DDX31	17085621	17861350	17582341	17509771	392923.3
DHX32	13381062	12940082	13491025	13270723	291574.3
DHX33	12458031	12391030	12041039	12296700	223929
DHX34	8758392	8572501	8406071	8578988	176250.1
DHX35	954445	949765	1064528	989579.3	64949.62
DHX36	17497413	17190038	17381048	17356166	155190.8
DHX37	25362143	26081046	25830027	25757739	364862.4
DHX38	8327631	8271037	8408327	8335665	68996.7
DHX39A	11296024	10870250	11093481	11086585	212970.8
DHX40	15381017	15690498	15780230	15617248	209444.2
DDX41	18028368	18242683	19236781	18502611	644776.9
DDX42	13260581	13104598	12910058	13091746	175614.6
DDX43	8625314	8347061	8730461	8567612	198106.1
DDX46	11506831	10972591	11382712	11287378	279588.1
DDX47	8231592	8410646	8045031	8229090	182820.3

EIF4A3	634745	625893	653926	638188	14330.14
DDX49	16809322	17130561	17281003	17073629	240939.2
DDX50	12658031	12480057	12710305	12616131	120707.3
DDX51	12510973	12170204	12693054	12458077	265408.2
DDX52	28075334	19247918	21412465	22911906	4600767
DDX53	10360728	10481037	10607139	10482968	123216.8
DDX54	10706289	9804102	10381024	10297138	456905.8
DDX55	15497021	16180451	15810472	15829315	342104.4
DDX56	14268023	13980257	14480205	14242828	250924.4
DHX57	11930827	11692037	12272051	11964972	291510.6
DDX58/RIG-I	7213624	7481048	7102631	7265768	194522.7
DHX58/LGP2	15370382	15971006	15710308	15683899	301181.7
DDX59	13285602	12786105	13105902	13059203	253001.8
DDX60	13008162	12491035	13370206	12956468	441859.3
MDA5	17805420	18326004	18092374	18074599	260746.8
siFLuc	586227	603872	598761	596286.7	9079.001
